# Cause and Possible Treatments of Foot Lesions in Captive Syrian Hamsters (*Mesocricetus auratus*)

**DOI:** 10.4061/2010/951708

**Published:** 2010-06-13

**Authors:** Mélisa Veillette, Julie Guitard, Stéphan G. Reebs

**Affiliations:** Département de Biologie, Université de Moncton, Moncton, NB, Canada E1A 3E9

## Abstract

Syrian hamsters (*Mesocricetus auratus*) run extensively in exercise wheels. This running may cause paw lesions. Three treatments of these wounds (topical application of vitamin E, wheel blocking, and a combination of both) were compared using both sexes. A pretreatment period with or without wheels lasted 15 days and the ensuing treatment period lasted 45 days. At the end of the pre-treatment period, none of the animals without wheels had paw wounds, whereas at least 75% of the females and 100% of the males with wheels did. Females had fewer and smaller wounds than males at this point. At the end of the treatment period, no effect of vitamin E could be discerned, but significant wound healing occurred after wheel blocking in both males and females. Wheel blocking is an easy way to prevent or treat paw wounds, but it presents problems in terms of animal welfare, as wheels are an important cage enrichment for hamsters.

## 1. Introduction

Syrian hamsters (*Mesocricetus auratus*) greatly use running wheels in captivity. They can run over 10,000 wheel revolutions in a single night [1–3]. This running may or may not be stereotypic, but it is voluntary and is probably a self-reinforcing behaviour [4, 5]. After young hamsters start running in wheels for the first time in our laboratory, lesions commonly appear on their paw pads or toes [2, 3, 6]. These paw wounds (which should not be confused with the skin lesions that sometimes develop on the side of legs when they slip between the bars of a wheel; see [7]) usually take the shape of small cuts or dots that rapidly scab over [3]. Some of them can be as much as a few millimeters long, but they do not prevent the hamsters from continuing to run in their wheels [3].

Paw lesions may form without wheel access in other rodents and in some cage types, but they take much longer to develop than when wheels are available. For example, rats take 400 days to develop paw wounds without wheels [8] whereas hamsters require only 10–15 days with wheels [3]. This points to running activity as the likely cause of the wounds, though this has never been formally tested through wound monitoring following wheel removal.

Beaulieu and Reebs [3] documented the effect of different bedding materials and wheel running surfaces on paw wound formation in hamsters. They found that males ran more than females, but that wounds were similar in both sexes. However, females tended to have more front paw wounds and their wounds tended to last longer. Hamsters on pine bedding had fewer wounds than on hardwood flakes. Also, hamsters with wheels covered in a plastic mesh took longer to develop wounds than on wheels with naked metal bars, though these wounds, once formed, were larger and persisted for longer. The researchers found that many of the wounds did not heal during the study period, and they recommended veterinary treatment. 

One such treatment could be vitamin E. The effect of topical applications of vitamin E in scar reduction has been studied in humans and rats, though conclusive findings about its effectiveness are still lacking [9–13]. However, its protection against UV damage to the skin is well established (review [9]).

The goal of this paper was first to confirm that wheel running is the cause of paw wound formation, and then to compare the effectiveness of a vitamin E treatment with the more obvious (but possibly less desirable in terms of animal welfare) alternative of blocking the running wheel. Because slight differences exist between males and females in running levels and paw wound formation [3], both sexes were tested.

## 2. Materials and Methods

All protocols were approved by the Université de Moncton animal care committee (protocol # 08-02) in accordance with the ethical guidelines established by the Canadian Council of Animal Care [14]. Syrian hamsters (25 males and 25 females) were purchased from Charles River Canada. They were all 60 days old at the start of the experiment and had had no prior experience with running wheels. Sexes were tested separately in time (females immediately after males) to prevent olfactory and acoustical influences on behaviour. Room temperature was set at 21°C and humidity levels were 45%–60%. Light intensity (fluorescent lights) was 1,000 lux. The light:dark cycle was 14 : 10 hours. This long photoperiod is commonly used in laboratory settings, most notably during chronobiological studies, as it keeps hamsters in their reproductive phase and therefore active. 

Each hamster was placed in a transparent polypropylene cage (42 × 22 × 21 cm, L × W × H). All cages were provided with pine shaving bedding (Canada Grade, 1 cm deep), distilled water, and food pellets (Pro Lab: Lab Diet) on the bottom of the cage. Depending on treatment, some cages had an exercise wheel (35 cm diameter, with stainless steel bars 9.5 cm long and 2 mm thick, 7 mm space between bars, Nalgene, F-size for rats). The Canadian Council of Animal Care [14] recommends the use of rat-size running wheels for hamsters, and this is the type normally used in our laboratory (see [6]). Wheel counts were noted daily but equipment failure prevented us from obtaining complete datasets, which in turn prevented us from quantifying the relationship between running intensity and wound severity beyond a wheel-no wheel dichotomy. The cages with no wheels were given wooden toys (Living World), a wooden platform, and a section of Habitrail tunnel (18 cm long, 6 cm in diameter) as compensatory cage enrichment. 

The five experimental groups were randomized by treatment and location in the room. Five animals of each sex were assigned to each of these five groups. The groups consisted of three treatments and two controls. 

The three treatment groups were exposed to a pretreatment period with wheels available until at least 70% of hamsters had at least one wound present on their paws, which took 15 days. This was followed by a treatment period which lasted 45 days (day 15 through day 60). The treatment consisted of either (a) blocking the wheel, (b) leaving the wheel free but applying vitamin E (Life Natural source, 1,000 IU, high purity) daily onto the wounds, or (c) blocking the wheel and applying vitamin E onto the wounds. The vitamin E was in a gel capsule, a drop of which was placed on the wound. This form was used because it is ingestible, an important consideration given that hamsters could lick their paws.

The two control groups were also followed for 60 days (the first 15 counting as a virtual pretreatment period and the next 45 counting as a virtual treatment period), but they never received treatment if and when paw wounds appeared. The first control group never had access to a running wheel, while the second control group always had a free wheel available. 

Paws were inspected once daily in all hamsters. During inspection, wounds were counted, the size of all of them was measured with a 15 cm ruler graduated in millimeters, and vitamin E was applied if appropriate for the experimental group. During paw inspection and treatment, hamsters were picked up by the skin of the neck and turned over in the surrender position [15]. 

One male hamster in the vitamin E treatment was removed from analysis as it died of unknown causes before the end of the experiment. Another male hamster from the wheel-always-present control group was removed from the experiment on day 44 (out of 60) because the wounds on his paw became a severe case of pododermatitis, but his data could still be used because the wound would not have healed and was given the maximum size it had had until then. These removals affected the degrees of freedom for the analysis of the 60-day time frame. 

Proportion of hamsters with at least one wound present, number of wounds per animal, and mean wound size were the response variables. The latter two were amenable to analysis with non-parametric ANOVAs, following rank transformations, to test for the effects of sex and group both at the end of the pretreatment period (day 15) and at the end of the treatment period (day 60). Parametric ANOVAs could not be used because conditions of normality and homogeneity of variance could not be met. A *t*-test was also used to test for weight differences between the sexes on day 60. Statistical analyses were performed using SAS version 9.1 (SAS Institute Inc.).

## 3. Results

**Figure 1:**
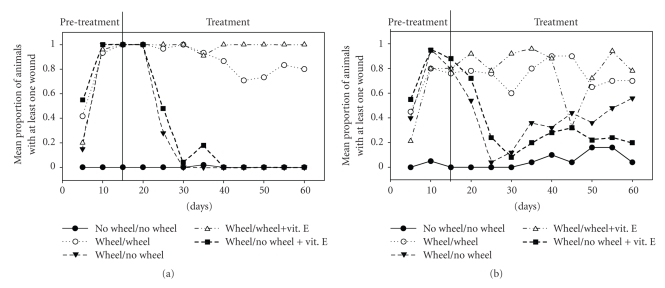
Proportion of male (a) and female (b) hamsters showing at least one paw wound during the experiment. Data points show the mean for the past five days and the five animals of each experimental groups, the names of which indicate their pretreatment and treatment conditions. “No wheel/No wheel” and “Wheel/Wheel” were the control groups.

Figure 1 shows the proportion of hamsters that had at least one wound present. In the pretreatment period, all the males that had access to a wheel developed at least one wound, whereas control males with blocked wheels were all wound-free (Figure 1(a)). After treatment started for the males, it took about 15 days for most of the wounds to heal in the two blocked-wheel experimental groups, whether or not vitamin E was daubed on their paw wounds, whereas only one of the males with free wheels healed completely, again irrespective of whether they had vitamin E or not. The results were similar for females, though somewhat less clear-cut (Figure 1(b)). At least 75% of the females with wheels showed at least one wound at the end of the pretreatment (rather than 100 % as in males), but as in males, all females without wheels were wound-free. Females that had free wheels during the treatment phase did not heal well, whereas those that had their wheels blocked healed in 15 days, though some developed a few new wounds thereafter (as opposed to males, who remained wound-free until day 60).

**Figure 2:**
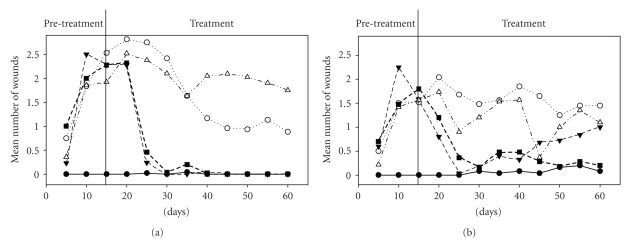
Mean (*n* = 5) number of wounds per animal in male (a) and female (b) hamsters during the experiment. Legend as in Figure 1.

Figure 2 shows the mean number of wounds per animal. The overall statistical model at 15 days (end of pretreatment) was significant (*F*_9,42_ = 5.44, *P *< .001, *r* ^2^ = 0.54) with significant effects of both sex (*F*_1_ = 4.47, *P* = .04) and group (*F*_4_ = 10.52, *P *< .001). Contrasts revealed that males had on average more wounds than females at day 15 (*P* = .04), and that the only group difference was between the no-wheel control and all the other groups that had free wheels (*P *< .001), reflecting the fact that all hamsters without wheels had no wounds at the end of the pretreatment period, whereas those with wheels had between 1.5 and 3 lesions on average (Figure 2). On day 60 (i.e., after 45 days of treatment), the overall model remained significant (*F*_9,39_ = 7.74, *P *< .001, *r* ^2^ = 0.64). However, only the group effect persisted (*F*_4_ = 14.78, *P *< .001), and there was an interaction between sex and group (*F*_4_ = 3.18, *P* = .02), with females having fewer wounds than males in the with-wheel groups, but more wounds in the non-wheel groups. Contrasts showed that hamsters with wheels still had more wounds than those without (*P *< .001). Also, animals that had their wheels blocked ended up with significantly fewer wounds than those that had vitamin E but that did not have their wheels blocked (*P* < .001, Figure 2).

**Figure 3:**
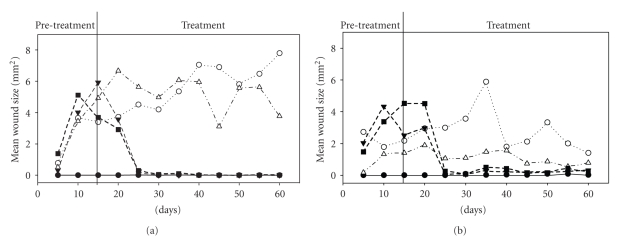
Mean (*n* = 5) wound size for male (a) and female (b) hamsters during the experiment. Legend as in Figure 1.

The same model with mean size of wounds (Figure 3) was also significant at day 15 (*F*_9,42_ = 4.58, *P* < .001, *r* ^2^ = 0.495) and followed the same trends as with the number of wounds. There were effects of both sex and group. Males had larger wounds than females at day 15 (*P* = .04) and wound size was obviously higher with the wheel than without (*P *< .0001), as there were no wounds without wheel access. There were no interactions, and no other group contrasts were significant at day 15. At the end of the treatment period on day 60, the overall model was again significant (*F*_9,39_ = 8.66, *P* < .0001, *r* ^2^ = 0.67). As with number of wounds the main effect of sex disappeared, but the effect of group remained (*P *< .0001). Hamsters had larger wounds in the with-wheel control and the with-wheel treatments than in the groups without wheels. There was also a nearly significant interaction of sex by group (*P* = .054): females had much smaller wounds than males in the wheel groups, but slightly larger wounds than males in the no-wheel groups (*P *< .0001, Figure 3). 

Vitamin E did not have curative effects. Contrasts showed no significant differences for neither the number of wounds nor wound size between the with-wheel control and the with-wheel + vitamin E treatment (*P* = .53 and *P* = .81 resp.; compare open circles and open triangles on Figures 2 and 3). Additionally, there was no significant difference between the no-wheel groups and the no-wheel + vitamin E treatment for either number of wounds or wound size (*P* = .15 and *P* = .46, resp.; compare closed triangles and closed squares on Figures 2 and 3).

Male and female weights were, respectively, 112.6 ± 3.6 g (mean ± S.D.) and 117.8 ± 3.9 g at the begining of the experiment, and 136.6 ± 7.8 g and 147.0 ± 11.9 g on day 60. The difference in weight between males and females on day 60 was statistically significant (*P* = .001).

## 4. Discussion

The results clearly show (formally for the first time) that wheel running is responsible for paw wound formation in hamsters. During pretreatment, none of the hamsters without wheels developed a single paw wound, whereas most of the animals with a wheel did. During treatment, hamsters that kept their wheels did not heal, whereas blocking the wheel resulted in almost complete healing within 15 days. In some females with blocked wheels the wound problem resurfaced after day 30, but to a lower degree than with the wheel in the pretreatment phase. This recurrence could have been caused by the females hanging and sometimes slipping from the hardware cloth that closed the top of their cages (personal observations). Very small (see Figure 3(b)) wounds appeared on the front paws of the females that did not have wheels, possibly caused by this climbing activity. That such wounds did not appear in males cannot be explained by differences in climbing tendency, as both sexes are known to climb equally [16], but may be related to the difference in weight between males and females, as females were on average heavier than males and may have slipped more.

No curative effect of vitamin E application was detected. However, the effectiveness of our application method may have been compromised by the fact that hamsters licked their paws as soon as they were placed back into the cage (personal observations) and thus they removed at least part of the daubed gel. The vitamin E treatment could be tested again with a wrapping or other means that would prevent the animals from licking their paws, at least temporarily. 

Because this experiment showed that the running wheel causes paw wounds, and that its blocking allows wound healing, it could be argued that removing the wheel entirely would be the treatment of choice. However, wheels are an important enrichment for hamsters and wheel running is a self-reinforcing behaviour [4, 16]. Our wheel count dataset, incomplete though it was, revealed averages of nearly 8,000 revolutions a night. Even the animal that was removed from the experiment due to excessive infection of one of its paws was still running in the wheel on its lame leg at the time (personal observations). If wheel blocking is used as a treatment for paw wounds, it will be important in terms of animal welfare to unblock the wheel as soon as the healing is complete, though of course wounds might then reappear.

A regime of partial daily wheel blocking combined with other cage enrichments as compensation may in fact be preferable, but this remains to be investigated. The relationship between paw wound occurrence and intensity of running is still unknown. The wheels given to hamsters in our laboratory are large, which is what the animals prefer [6], and consequently they run intensively. If paw wounds form only because of very high running, then partial wheel blocking might help alleviate or prevent wound formation without greatly compromising the animals' welfare.

## 5. Conclusions

Wheel running is the cause of paw wounds in hamsters, and blocking the running wheel is an effective treatment for paw wounds. Daily topical application of a vitamin E gel had no effect on wound occurrence, size, and number, though the effectiveness of the gel may have been mitigated by the animals licking at least part of the gel off their paws. A recommendation can be made to block the wheel for several weeks (15–20 days) after paw wounds appearance, though animal welfare and cage enrichment considerations dictate the option of limiting this treatment to the most extreme cases, or perhaps using a schedule of partial daily blocking only.
